# Additivity vs. Synergism: Investigation of the Additive Interaction of Cinnamon Bark Oil and Meropenem in Combinatory Therapy

**DOI:** 10.3390/molecules22111733

**Published:** 2017-11-04

**Authors:** Shun-Kai Yang, Khatijah Yusoff, Chun-Wai Mai, Wei-Meng Lim, Wai-Sum Yap, Swee-Hua Erin Lim, Kok-Song Lai

**Affiliations:** 1 Department of Cell and Molecular Biology, Faculty of Biotechnology and Biomolecular Sciences, Universiti Putra Malaysia, 43400 Serdang, Selangor, Malaysia; kaichan992@gmail.com; 2 Department of Microbiology, Faculty of Biotechnology and Biomolecular Sciences, Universiti Putra Malaysia, 43400 Serdang, Selangor, Malaysia; kyusoff@upm.edu.my; 3 Perdana University-Royal College of Surgeons in Ireland, Perdana University, MAEPS Building, Serdang, Selangor, Malaysia; erinlim@perdanauniversity.edu.my; 4 Department of Pharmaceutical Chemistry, School of Pharmacy, International Medical University, No. 126, Jalan Jalil Perkasa 19, Bukit Jalil, 57000 Kuala Lumpur, Malaysia; chunwai_mai@imu.edu.my; 5 Department of Pharmaceutical Technology, School of Pharmacy, International Medical University, No. 126, Jalan Jalil Perkasa 19, Bukit Jalil, 57000 Kuala Lumpur, Malaysia; weimeng_lim@imu.edu.my; 6 Department of Biotechnology, Faculty of Applied Sciences, UCSI University, 56000 Cheras, Kuala Lumpur, Malaysia; wsyap@ucsiuniversity.edu.my; 7 Health Sciences Division, Abu Dhabi Women’s College, Higher Colleges of Technology, 41012 Abu Dhabi, United Arab Emirates

**Keywords:** additive interaction, antibiotic resistance, cinnamon bark essential oil, combinatory treatment, membrane disruption

## Abstract

Combinatory therapies have been commonly applied in the clinical setting to tackle multi-drug resistant bacterial infections and these have frequently proven to be effective. Specifically, combinatory therapies resulting in synergistic interactions between antibiotics and adjuvant have been the main focus due to their effectiveness, sidelining the effects of additivity, which also lowers the minimal effective dosage of either antimicrobial agent. Thus, this study was undertaken to look at the effects of additivity between essential oils and antibiotic, via the use of cinnamon bark essential oil (CBO) and meropenem as a model for additivity. Comparisons between synergistic and additive interaction of CBO were performed in terms of the ability of CBO to disrupt bacterial membrane, via zeta potential measurement, outer membrane permeability assay and scanning electron microscopy. It has been found that the additivity interaction between CBO and meropenem showed similar membrane disruption ability when compared to those synergistic combinations which was previously reported. Hence, results based on our studies strongly suggest that additive interaction acts on a par with synergistic interaction. Therefore, further investigation in additive interaction between antibiotics and adjuvant should be performed for a more in depth understanding of the mechanism and the impacts of such interaction.

## 1. Introduction

Antimicrobial resistance is an ongoing challenge in the clinical setting at present, mainly due to a lack of compliance by patients and health practitioners, coupled with extensive and overuse of antibiotics in the agriculture and aquaculture sectors [1]. This phenomenon has triggered the demand for continuous discovery and commercialization of applications for novel, yet effective new antimicrobial agents. However, the limiting factor that remains is the rate of new drug development versus the rate of bacterial evolution, with bacterial evolution emerging as the undisputed winner [2]. Several strategies have been proposed to tackle this issue, including newer drug rotation and combination therapies. The latter is the preferred strategy as drug rotation can only be applied when there is an extensive availability of novel antimicrobials.

Currently, only two forms of combinatory therapies have been applied in clinical settings to tackle multi-drug resistant bacterial infections; antibiotic-antibiotic combination and antibiotic-adjuvant combination [3]. As the name suggests, antibiotic-antibiotic combination involves the use of different classes of antibiotics, with the most common combinations being β-lactams and aminoglycosides or fluoroquinolone. However, this combinatory treatment is not recommended as it would worsen the issue of antibiotic resistance in bacteria [4]. The second option, however, involves the use of an adjuvant, a compound which, when administered alone has minimal or no antimicrobial activity, but increases the effectiveness of the antibiotic synergistically when used in combination as a whole. A common examples of antibiotic-adjuvant combination used is a β-lactam antibiotic (amoxicillin) with a β-lactamase inhibitor (clavulanic acid) [3]. In addition, countless studies have been carried out, with the mining of new antimicrobial agents for use in combinatory treatment being expedited with the hopes of addressing the prevalent antibiotic resistant superbug. In addition, with the general public becoming more health conscious, the mining of novel antimicrobials have also shifted its direction from synthetic chemical compounds to greener plant-based compounds such as essential oils, considering the relatively reduced adverse effects and cost effectiveness of natural products, when developed at a commercial scale [5].

Drug interaction in combinatory treatments can be divided into three classifications: synergistic, additive and antagonistic. The synergistic approach, whereby the combined action of both agents is more effective than the action of single agent, has been the focus of most combination treatments as this interaction type has understandably been known to be the most effective. Additivity, on the other hand, is less preferred compared to synergism wherein the interaction between two drugs is mutually exclusive to each other, resulting in lesser significant reduction of applied dosages when used singly or combined. Finally, antagonism occurs when one of the agents used counteracts the action of the other agent, reducing the effectiveness of any one of the agents when used alone [6]. An antagonistic interaction is preferred in downplaying the adverse effects that one drug may possess while enabling it to still exert its desired effect. The determining factor for the relationship between antimicrobial agents (synergistic, addictive or antagonistic) lies in the fractional inhibitory index obtained via the checkerboard assay, whereby indices lower than 0.5 are synergistic, indices in between 0.5 and 4 are additive and indices greater than 4 are antagonistic in nature [7,8]. 

For many years, synergism has been the main focus for combinatory therapy as it greatly reduces the effective dosage of the antimicrobial agents required to treat an infection. However, the effects of additivity, that can also lower the minimal effective dosage of either antimicrobial agent, had been sidelined from combinatory therapy due to the threshold indices which limits synergistic interaction under a narrow gap of indices [8]. Thus far, only synergistic combination (FICIc ≤ 0.5) has been the major focus for downstream investigation such as determination of mode of action and toxicity evaluation. It was reported by Si et al. that amongst 11 combinations of antibiotics and oregano essential oil, only five showed synergismm while the other combinations reacted additively [9]. Later, the study performed by van Vuuren et al. obtained 25 synergistic essential oil-antibiotic combinations among the 72 tested; 65% of the combinations showed additivity [10]. Karpanen et al. also reported that all four combinations of thymol, a plant secondary metabolite, in combination with antibiotics only reacted additively when used against multidrug resistant *Staphylococcus aureus* (MRSA) [11]. Another antimicrobial screening against MRSA, performed by Chovanová et al. yielded that only 50% of the screened plant extracts exhibited synergism with the antibiotic oxacillin; the other 50% tested plant extract interacted additively to oxacillin [12]. In addition, in a study performed by Yap et al. whereby a total of 35 combinations of essential oils and antibiotics were screened for their synergistic capabilities against multi-drug resistant *Escherichia coli,* only five combinations showed synergism while the other 30 combinations interacted additively [13]. Some of these synergistic combinations had been subjected to downstream mechanism analysis, but none has yet ventured into clinical testing, due to high fractional concentrations of the synergistic compounds which are not suitable for clinical application [14]. Of the five combinations reported in [13], only two combinations were further investigated for their modes of action [15,16]. 

Over the years, there have been a number of additive combinations of crude extracts or individual compounds with antibiotics which have been investigated but remained untapped. Furthermore, no detailed studies had been carried out to evaluate and compare actual additive interactions between adjuvant and antibiotics. Hence, investigation in this area would open up new possibilities whereby new combinations of adjuvant and antibiotics can be established, further reducing the severity of antimicrobial resistance in pathogens. Although additivity interactions may not be as effective as synergistic interactions; the concentration of the adjuvant needed to achieve additivity in combinatory treatment might be lower than what is observed in synergistic interactions. This would significantly increase the chances of downstream analysis to the point of even clinical trials being performed. Thus, additivity interaction in antibiotic-adjuvant therapy deserves further investigation as they may be applicable in the clinical setting, despite a less robust effect to inevitably, exert a lesser degree of adverse outcome. Therefore, this study aims to look at the effect of additivity between essential oils and antibiotics, via the use of cinnamon bark essential oil (CBO) and meropenem as a model for additivity.

## 2. Results

### 2.1. Resazurin Microplate Assay and Checkerboard Assay

All four tested essential oils exhibited additive interactions with meropenem against *K. pneumoniae* BAA-1705 with CBO-meropenem giving the highest FICIc value, 1.00 which is two fold the FICIc value of the other combinations (Table 1). However, tea tree oil, when combined with meropenem successfully reduced the dosage of meropenem from 32 μg/mL to 0.5 μg/mL, a 64-fold reduction. In order to further investigate the effects of additivity in combinatory therapy, CBO and meropenem combination was selected based on the highest FICIc as a good representation of additivity in subsequent assays. 

### 2.2. Time Kill Analysis

**Figure 1:**
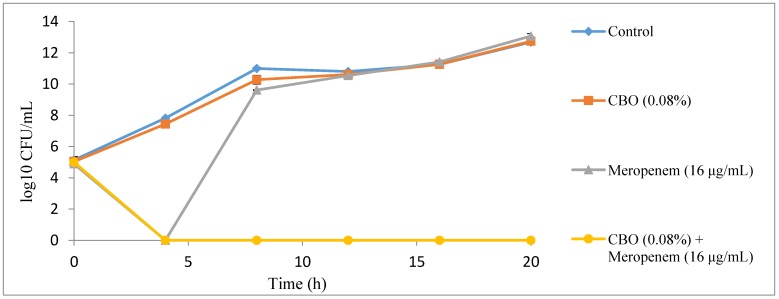
A 4-h interval killing curve for CBO and meropenem alone, and in combination against *K. pneumoniae* BAA-1705.

**Figure 2:**
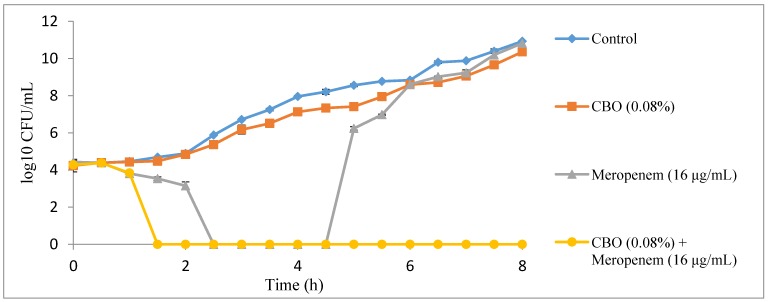
A 30-min interval killing curve for CBO and meropenem alone, and in combination against *K. pneumoniae* BAA-1705.

In the time kill analysis, a complete killing profile of *K. pneumoniae* BAA-1705 treated with combination of CBO and meropenem was observed at 4 h at the preliminary time kill analysis which had 4 h interval of viable counting time, lasting up to 20 h (Figure 1). Then, the time kill analysis was repeated by shortening the viable counting time to every 30 min until the 8th hour. It was observed that only 1.5 h were required to obtain a complete killing profile for *K. pneumoniae* BAA-1705 treated with combination of CBO and meropenem (Figure 2). Sub-inhibitory concentration of CBO (0.08%) alone was only able to insignificantly lower the growth of the bacteria. Conversely, the sub-inhibitory concentration of meropenem (16 μg/mL) alone inhibited the growth of the cells for the first 2.5 h as shown in Figure 2. At the 5th hour, however, the cells continued to grow exponentially with no significant difference between the control and the cells treated with CBO alone. 

### 2.3. Zeta Potential Measurement

**Figure 3:**
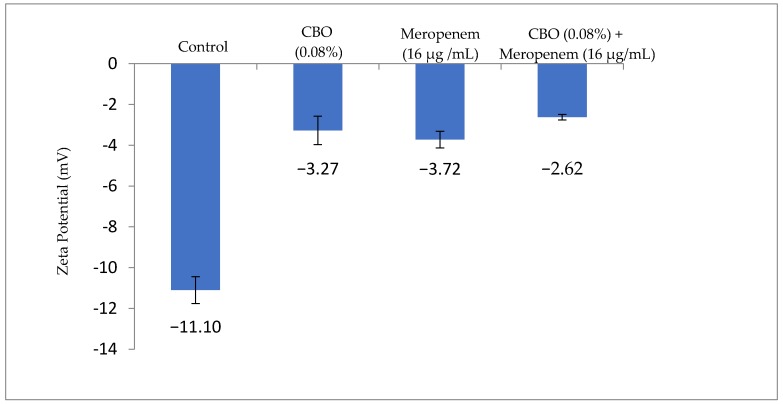
Zeta potential value (mV) of *K. pneumoniae* BAA-1705 treated with CBO and meropenem, alone and in combination.

Following the 5 h treatment time obtained from the time kill analysis, the bacterial surface charge was determined using the zeta potential measurement to detect the mobility of bacterial cells in the presence of an electrophoretic force, given that the pH and salt concentration is standardized. According to Figure 3, the zeta potential of the non-treated *K. pneumoniae* BAA-1705 has a negative value of −11.1 mV while treated cells had significantly more positive values of −2.62 mV to −3.27 mV. Individually treated *K. pneumoniae* BAA-1705 cells with either CBO or meropenem alone had zeta potential value of −3.27 mV and −3.72 mV, respectively, whereas the combination of both had the most positive zeta potential value, −2.62 mV (Figure 3).

### 2.4. Outer Membrane Permeability Assay

**Figure 4:**
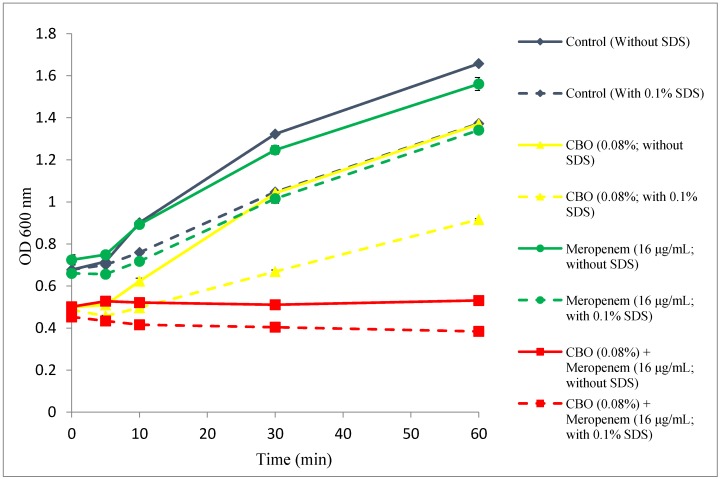
Comparative absorbance of treated *K. pneumoniae* BAA-1705 subjected to 0.85% saline solution and 0.1% SDS influx.

Figure 4 compares the growth of the *K. pneumoniae* BAA-1705; control (only MHB broth supplemented with 10% Tween 80), treated with CBO (0.08%) and meropenem (16 μg/mL) alone and in combination, in terms of absorbance at 600 nm between pre- and post-exposure to 0.1% SDS solution. 

The control group and cells treated with sub-inhibitory concentration of meropenem showed normal growth in the absence of SDS with the control group having higher growth rate than cells treated with meropenem. In the presence of SDS, there was a reduction in the growth rate as even a low concentration of SDS (0.1%) exerts stress to the growing cell. However, bacterial cells treated with CBO alone and combination of CBO and meropenem, in the absence or presence of 0.1% SDS, had lower absorbance compared to those of controls and treated with meropenem, indicating that CBO exhibit more significant inhibitory effects than meropenem. A significant drop in the absorbance can be detected when CBO treated cells were exposed to 0.1% SDS, indicating the ability of CBO to cause sudden influx of SDS into the cell, and halted cell growth. Combination of CBO and meropenem had the lowest absorbance reading, with a decreasing trend, among four groups, especially when exposed to 0.1% SDS.

### 2.5. Scanning Electron Microscopy

**Figure 5:**
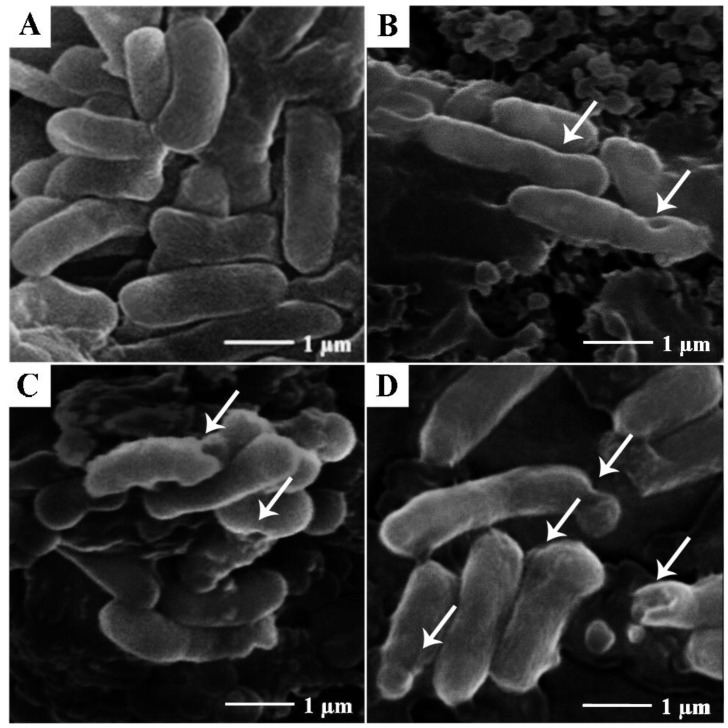
Scanning electron micrograph of *K. pneumoniae* BAA-1705. (**A**) Non-treated cells; (**B**) cells treated with CBO (0.08%); (**C**) cells treated with meropenem (16 µg/mL); (**D**) cells treated with combination of CBO (0.08%) and meropenem (16 µg/mL).

In order to determine the morphological changes of *Klebsiella pneumoniae* BAA-1705 after treatment with CBO (0.08%) and meropenem (16 μg/mL), alone and in combination, we conducted scanning electron microscopy studies on these bacteria. As shown in Figure 5A, non-treated *K. pneumoniae* BAA-1705 were typical rod-shaped bacteria with smooth surfaces. Upon exposure to CBO at 0.08%, irregularities in the morphology of the bacteria was observed, with the presence of openings in the cell envelope, as indicated by arrows in Figure 5B. 

Similarly, *K. pneumoniae* BAA-1705 treated with meropenem (16 µg/mL) also showed minor corrugation and irregularities on the surface membrane of the bacteria (Figure 5C). Last but not least, combinatory treatment with both CBO (0.08%) and meropenem (16 µg/mL) produced the most extensive distortion and corrugation in the cell envelope of the bacteria.

## 3. Discussion

Initially, a collection of essential oils were screened for their combinatory effects with meropenem. Four pairs of combinations which indicated additive interaction, namely, cinnamon bark, marjoram, peppermint and tea tree essential oils with meropenem were presented in this study. Other pairs exhibiting synergistic interactions were excluded from this study as the main focus was to investigate and compare the application of additivity in antibiotic combinatory therapy. As seen from Table 1, marjoram, peppermint and tea tree reduced the inhibitory concentration of meropenem from 32 μg/mL to 2 and 0.5 μg/mL respectively. On the other hand, cinnamon bark oil (CBO) only managed to reduce the inhibitory concentration of meropenem from 32 μg/mL to 16 μg/mL. The effectiveness of the reduction in the inhibitory concentration lies in the outer membrane disruptive ability of essential oils as influx of meropenem is only facilitated by the outer membrane porin or a disturbance in the permeability of the outer membrane [18]. This shows that CBO had lower membrane disruptive ability when compared with the other three essential oils. From these four pairs of essential oils and meropenem, CBO was selected for subsequent assays as it had the highest fractional inhibitory index (FICIc = 1), compared to the other essential oils which had FICIc values closer to synergistic interaction (0.52 and 0.56), this would reflect and represent the effects of additivity instead of synergistic in this study. In a similar study, Yap et al. showed that CBO in combination with a variety of antibiotics gave the highest degree of additivity when used against multi-drug resistant *E. coli* [13]. The fractional inhibitory concentration or sub-inhibitory concentration CBO (0.08%) and meropenem (16 µg/mL) obtained from the checkerboard assay were used in subsequent assays to make comparison between the effects of agents alone and in combination. 

In order to determine the optimum time required to inhibit the growth of *K. pneumoniae* BAA-1705 by the combination of CBO and meropenem, time kill analysis was performed on all four treatment groups; control, treated with CBO alone, treated with meropenem alone and treated with the combination of CBO and meropenem. Synergism and antagonism between two antimicrobial agents can also be detected via time kill analysis. Synergistic interaction is interpreted as a decrease of more than 2 log_10_ CFU/mL in the combinatory treatment group when compared to either single agent treatment group whereas an increase of more than 2 log_10_ CFU/mL indicates antagonism [19]. As observed in Figure 1 and Figure 2, combinations of CBO and meropenem at their respective sub-inhibitory concentrations resulted in a decrease of more than 2 log_10_ CFU/mL when compared to those treated with CBO and meropenem alone, thus indicating synergism. Conversely, data obtained via the checkerboard assay suggests that the interactions between CBO and meropenem, are additive, which suggests that time kill analysis is merely an assay that is suitable in the determination of the killing time, but not for differentiating synergistic, additive and antagonistic interaction between compounds. In order to determine the relationship between two antimicrobial agents, an additional method such as the checkerboard assay and the E test method, which are more reliable, should be coupled with time kill analysis [7,20,21]. A sudden drop in the number of bacterial colonies treated with sub-inhibitory concentration of meropenem detected at 4th hour in Figure 1 and from 2.5 to 4.5 h was due to the lag phase in bacterial growth. The presence of meropenem, even in its sub-inhibitory concentration, exerted significant stress which temporarily inhibited the growth of the bacteria. Furthermore, the weakened bacteria were unable to grow when plated on MH agar plate as solid media provide another challenge for proper growth in their weakened state [22]. Thus, this explains the absence of colonies at the mentioned time point. Normal growth was resumed at the 5th hour as *K. pneumoniae* BAA-1705 treated at sub-inhibitory concentration of meropenem alone had developed enough carbapenemase to hydrolyze the existing meropenem molecules which was transported into the periplasmic space via outer membrane porin, preventing meropenem from permanently acylating and deactivating the penicillin binding protein (PBP) [18]. In contrast, no growth was observed in the bacteria culture treated with the combination of CBO and meropenem at their respective sub-inhibitory concentration due to the postulated mode of action of CBO in interfering the bacterial membrane stability and increasing the permeability of outer membrane, which, eventually facilitates the influx of meropenem as previously reported by Yap et al. (2015) and Zhang et al. (2016) [15,23].

Subsequently, zeta-potential measurements, outer membrane permeability assays and scanning electron microscopy studies were performed to further understand and confirm the role of CBO in interfering bacterial membrane stability and permeability. Furthermore, the results from each assay were compared with those performed in other studies involving synergistic combination of CBO and antibiotics. The zeta potential measurement basically reflects bacterial metabolic state and membrane potentials; the higher the growth rate of bacteria, the more negative the measurement is [24]. As shown in Figure 3, cells within the control group had the most negative membrane potential whereas a drastic increment up to 3-fold can be seen in cells treated with CBO and meropenem alone and in combination. In comparison with the work of Yap et al. multi-drug resistant *E. coli* treated with synergistic combination of CBO essential oil and piperacillin alone and in combination had increased membrane potential by 2-fold when compared to the control group, suggesting that additivity interaction between CBO and meropenem demonstrated a more significant membrane disruption ability in comparison with a synergistic pairing [16]. The mechanistic action of CBO in membrane potential was reported by Yunbin and colleagues, bacteria treated with CBO displayed a 3- to 5-fold increment in their membrane potential when compared with non-treated bacterial cells [25]. 

To verify the integrity of the bacterial membrane, the outer membrane permeability test was carried out using 0.1% SDS as a permeabilizing probe. Under physiological circumstances, bacterial cells with functional outer membranes had the ability to prevent low concentrations of SDS from reaching the intracellular region of the cell, preventing cell lysis. However, in the presence of a permeabilizer, which disrupts the membrane permeability, an influx of SDS would occur, accumulating to a certain concentration and thus causes cell lysis [23,26]. The concentration of SDS was optimized and fixed at 0.1% as this concentration does not cause significant damage to bacterial cell with healthy outer membrane. In addition, the duration of this experiment was also optimized and fixed at 1 h in order to prevent cell damage due to prolonged exposure to SDS. Overall, the presence of CBO at 0.08% (alone and in combination with meropenem) significantly altered the outer membrane permeability, causing influx of SDS and eventually lysing the cell. This can be observed in the decreased OD when exposed to 0.1% SDS in Figure 4. According to a study conducted by Yap et al. which investigated the synergistic effects of CBO with piperacillin against multi-drug resistant *E. coli*, the OM permeability assay performed showed reproducible results whereby the presence of CBO aids in the influx of SDS which is normally excluded under normal circumstances [15]. Another similar study which looked at the synergistic relationship between lavender oil and piperacillin also showed an identical trend whereby the presence of essential oil causes the influx of SDS leading to cell death [16]. This further validates that synergism and additivity, perhaps shared similar severity in terms of bacterial membrane disruption.

Scanning electron microscopy was performed on all four groups of cells to observe the effect on membrane integrity of additive interaction between CBO and meropenem (Figure 5). In the presence of CBO alone, bacterial membranes were corrugated and deformed. However, the addition of meropenem caused a more severe corrugation and deformation to the cell morphology. Comparable observations were obtained from other studies involving synergistic combination of essential oil and antibiotic [15,16,27]. However, the scanning electron microscopy was only able to access the membrane disruption ability via qualitative means. Thus, comparison between synergism and additivity was further quantified and validated via zeta potential measurement and outer membrane permeability assay mentioned above. 

Additivity interactions do have its advantages; in this case, very low concentrations of CBO are required to achieve additivity with meropenem. As reported by Yap et al. essential oils responsible for synergistic interaction with antibiotics requires concentration at a range of 0.08% to 1%, but as shown in this study, only 0.08% of CBO and 0.6% of MO, PO and TTO is sufficient to achieve additivity [13]. In addition, according to the additive combination reported in this study, CBO with the highest degree of additivity at 0.08% had the ability to reduce the effective dosage of meropenem by 2-fold while other reported essential oils are effective at 0.63% resulting in a lower degree of additivity had reduced the effective dosage of meropenem by 16- to 64-fold in comparison. This suggests that, even in their additive state, only low concentrations of MO, PO and TTO is required to reduce the effective dosage of meropenem significantly and a similar observation have been observed in other reports mentioned earlier [9,10,11,12,13]. Additionally, evidence of low concentration used in a crude extract, such as essential oil indicates that the dosage of the individual or group compound within the essential oil needed to achieve additivity is even lower than the predecessor. Essential oils such as CBO are generally regarded as safe by the U.S. Food and Drug Administration, however, high dosages of essential oil would still cause toxicity in humans [28,29]. Thus it is important that the adverse effects of essential oils be investigated before clinical trials. This can be done via identifying the exact compound/s which is/are responsible for the additive interaction with antibiotics, thus eliminating compounds which play no role in such an interaction. As such, toxicity evaluations would be performed on minimal numbers of compounds instead of the crude essential oil having a larger variety of compounds that might contribute to the overall toxicity. Such evaluations would eventually, pave the way into the application of antibiotic-adjuvant combinatory treatment in the clinical setting. 

## 4. Conclusions

In conclusion, our study showed that additivity and synergistic interaction between CBO and antibiotic were comparable in their ability to cause bacterial membrane disruption, via comparison with similar studies carried out previously. Quantitative assessment of the membrane disruption ability between CBO and meropenem via zeta potential measurement further confirmed additivity transcended the synergistic combination between CBO and piperacillin. In addition, the outer membrane permeability assay and scanning electron microscopy studies carried out indicated comparable effects of additivity and synergistic interaction between CBO and antibiotics. Note should be taken our current study focuses particularly on the CBO essential oil, and this should not be assumed to represent the additive interaction as a whole. However, this preliminary evidence of the significance of additive interaction could be extrapolated to include other essential oil-antibiotic combination as a viable alternative to be supplemented with other treatment strategies in tackling antibiotic resistance. The additive interaction may not be able to substitute synergistic interactions completely; however, this could be a stimulus in propelling the efficacy of combinatory therapy in a new frontier. Perhaps in the future, additivity may actually transcend synergism. 

## 5. Materials and Methods

### 5.1. Essential Oils and Meropenem

Cinnamon bark (*Cinnamomum verum*), marjoram (*Origanum majorana*), peppermint (*Mentha x piperita*) and tea tree (*Melaleuca alternifolia*) essential oils used throughout the studies were purchased from Aroma Trading Ltd. (Milton Keynes, UK). Meropenem was purchased from Sigma-Aldrich Corporation (St. Louis, MO, USA) and dissolved in water to make a concentrated 10 mg/mL stock solution.

### 5.2. Bacterial Strains and Growth Conditions

The bacterial strains used in this study were *Klebsiella pneumoniae* BAA-1705 and *Escherichia coli* ATCC 25922, both obtained from American Type Culture Collection (ATCC, Manassas, VA, USA). Both bacterial strains were grown on Mueller-Hinton agar (MHA; Sigma-Aldrich). Subsequently, a single colony was inoculated into Mueller-Hinton broth (MHB; Sigma-Aldrich) at 37 °C and shaking at 250 rpm for 16 h. 

### 5.3. Resazurin Microplate Assay

The Resazurin Microplate Assay (REMA) was performed to determine the minimum inhibitory concentration (MIC) values of essential oils and meropenem of bacterial strains studied via broth microdilution as detailed in CLSI M07-A8. However, Tween 80, at final concentration of 10%, was incorporated into the MHB in order to enhance the solubility of cinnamon bark essential oil (CBO) whereas resazurin (7-hydroxy-3*H*-phenoxazin-3-one-10-oxide), at a final concentration of 0.02% was used to improve the visualization. Two-fold dilutions were performed in each test well to yield final well volumes composed of 50 μL of test compound, 40 μL of bacterial suspension at approximately 1 × 10^5^ cfu/mL and 10 μL of resazurin at a final concentration of 0.02%. *E. coli* ATCC 25922 was used for antibiotic (meropenem) positive control in this assay apart from negative and growth controls. All assays were performed in triplicates and incubated at 37 °C with shaking at 200 rpm for 20 h. The MICs of essential oils and meropenem were determined qualitatively and quantitatively via the color change in resazurin and relative fluorescent unit. 

### 5.4. Checkerboard Assay

The checkerboard assay was performed as detailed by Lorian with slight modifications, as described in the REMA [7]. Ten serial, two-fold dilutions of meropenem and five serial, two-fold dilutions of essential oils were prepared to determine the combinatory effects of essential oils and meropenem against *K. pneumoniae* BAA-1705. Each well contained 25 μL of meropenem and 25 μL of essential oils inoculated with 40 μL of bacterial suspension and 10 μL of resazurin to make final concentration of approximately 1 × 10^5^ cfu/mL and 0.02%. The 96-well plates were then incubated at 37 °C with shaking at 200 rpm for 20 h. Combinatory relationship between CBO and meropenem was expressed in terms of fractional inhibitory concentration index (FICI) using the following formulas [6,8]:

(1) $$\;\mathrm{FICI}\;\mathrm{of}\;\mathrm{CBO}=\;\frac{\mathrm{MIC}\;\mathrm{of}\;\mathrm{CBO}\;\mathrm{in}\;\mathrm{combination}}{\mathrm{MIC}\;\mathrm{of}\;\mathrm{CBO}\;\mathrm{alone}}$$

(2) $$\mathrm{FICI}\;\mathrm{of}\;\mathrm{meropenem}=\;\frac{\mathrm{MIC}\;\mathrm{of}\;\mathrm{meropenem}\;\mathrm{in}\;\mathrm{combination}}{\mathrm{MIC}\;\mathrm{of}\;\mathrm{meropenem}\;\mathrm{alone}}$$

(3)  FICIc=FICI of CBO+FICI of meropenem

FICIc ≤ 0.5, synergistic; FICIc > 0.5–4.0, additive; FICIc > 4.0, antagonistic.

The essential oil which yielded the highest value of FICIc in the additive range was used for subsequent assays.

### 5.5. Time Kill Analysis

A standard inoculum of 1 × 10^5^ cfu/mL was used in the time kill analysis via viable colony forming unit counting. The test concentration of essential oil and meropenem used were determined from the checkerboard assay with combination yielding the highest FICI in the additive range, among the four tested essential oil, which is cinnamon bark essential oil (CBO). The time kill analysis consisted of a non-treated control sample (inoculum with MHB supplemented with 10% Tween 80 at final concentration), samples treated with CBO (0.08%), samples treated with meropenem (16 μg/mL) and sample treated with the combination of CBO and meropenem (0.08% and 16 μg/mL). Each treatment had a final volume of 20 mL with Tween 80 incorporated at final concentration of 10% to enhance the solubility of CBO. Samples were incubated at 37 °C with shaking at 200 rpm. Immediately after inoculation, viable counting was performed every four hourly until 20 h. In the event of rapid killing, the measurement for viable counting was recorded every half hourly. A volume of 50 μL of samples were obtained and subjected to hundred-fold dilution with 0.85% (*w*/*v*) saline. Diluted samples were then plated onto Mueller-Hinton agar (MHA) and incubated at 37 °C for 16 h. The time kill analysis was performed in triplicates.

### 5.6. Zeta Potential Measurement

The zeta potential of non-treated and treated *K. pneumoniae* BAA-1705 cells (with CBO or meropenem alone, and in combination) were determined via a Zetasizer Nano ZS instrument (Malvern Instruments, Malvern, UK) [30]. The treatment time for all the treatment groups were as determined in the time kill analysis whereas the concentration of CBO and meropenem used were as determined from the checkerboard assay. Treated cells were washed with 0.85% saline for at least 5 times before zeta potential measurement. The experiment was performed in triplicates. 

### 5.7. Outer Membrane Permeability Assay

Overnight culture of *K. pneumoniae* BAA-1705 was washed, adjusted to optical density (OD_600nm_) 0.3 and then subjected to treatment by CBO and meropenem alone, and in combination with concentration and treatment time as determined via the checkerboard assay and time kill analysis. Once treatment was completed, the samples were first washed with 0.85% saline (five times) in order to remove the treatment, and then divided into two equal portions of 10 mL. Next, sodium dodecyl sulfate (SDS) solution at final concentration of 0.1% was added to one of the portion while 0.85% saline was added to the other. SDS acts as a permeabilizing probe that causes cell death when sudden influx occurred. This can be measured in terms of OD_600nm_ at intervals of 0, 5, 10, 30 and 60 min via a spectrophotometer [31,32]. The assay was completed in triplicates. 

### 5.8. Scanning Electron Microscopy

The final concentration and treatment time for CBO and meropenem, in combination and alone, were determined from the results of checkerboard assay and time kill analysis. The cells were harvested and the pellet was washed with 0.85% (*w*/*v*) saline for five times. Following that, the samples were then fixed with 4% glutaraldehyde for 5 h and 1% osmium tetroxide for 2 h at 4 °C. Sodium cacodylate buffer at 0.1 M was used in all the washing steps. Then, the samples were further dehydrated via sequential exposure to increase concentrations of acetone (35–95%) for 10 min followed by 100% acetone for 15 min for three times [19]. After dehydration, the samples were subjected to critical point drying for 30 min (BalTec CPD 030, Bal-Tec, Balzers, Liechtenstein). The samples were then secured onto the specimen stub using double sided tape. Finally, the samples were sputter-coated with gold using a cool sputter coater (BalTec SCD 005) and observed via a JEOL JSM-6400 instrument (JEOL, Tokyo, Japan) at 15 kV.

## Figures and Tables

**Table 1:** Minimum inhibitory concentration (MIC) and FIC indices of essential oil-meropenem pairs against *K. pneumoniae* BAA-1705.

Combinations of Essential Oils and Meropenem	*K. pneumoniae* BAA-1705				Type of Interaction
	MIC_O_	MIC_C_	FICI	FICIc	
Cinnamon Bark-Meropenem Cinnamon Bark (%)	0.16	0.08	0.50	1.00	Additive
Meropenem (μg/mL)	32	16	0.50		
Marjoram-Meropenem Marjoram (%)	1.25	0.63	0.50	0.56	Additive
Meropenem (μg/mL)	32	2	0.06		
Peppermint-Meropenem Peppermint (%)	1.25	0.63	0.50	0.56	Additive
Meropenem (μg/mL)	32	2	0.06		
Tea Tree-Meropenem Tea Tree (%)	1.25	0.63	0.50	0.52	Additive
Meropenem (μg/mL)	32	0.5	0.02		

MIC_O_, MIC of one sample alone; MIC_C_, MIC of one sample in the most effective combination. FICIc, total FIC of the combination of both sample, FICIc ≤ 0.5, synergistic; FICIc > 0.5–4.0, additive; FICIc > 4.0, antagonistic [8,17].

## Appendix A. Raw Data

**Table A1:** Raw data of time kill analysis at 4 h interval for 20 h.

Time (h)	*K. pneumoniae* BAA-1705			
	Cell Number (CFU/mL) (Mean ± SD)			
	Control	CBO (0.08%)	Meropenem (16 µg/mL)	CBO (0.08%) + Meropenem (16 µg/mL)
0	1.49 × 10^5^ ± 7.28 × 10^4^	1.10 × 10^5^ ± 1.41 × 10^4^	7.90 × 10^4^ ± 1.27 × 10^4^	1.00 × 10^5^ ± 1.41 × 10^4^
4	6.60 × 10^7^ ± 8.49× 10^6^	2.90 × 10^7^± 1.27 × 10^7^	1.00 × 10^1^ ± 0.00 × 10^0^	1.00 × 10^1^ ± 0.00 × 10^0^
8	9.95 × 10^10^ ± 1.48 × 10^10^	2.10 × 10^10^± 1.27 × 10^10^	4.15 × 10^9^ ± 2.12 × 10^8^	1.00 × 10^1^ ± 0.00 × 10^0^
12	6.35 × 10^10^± 7.78 × 10^9^	4.25 × 10^10^ ± 1.48 × 10^10^	3.55 × 10^10^ ± 9.19 × 10^9^	1.00 × 10^1^ ± 0.00 × 10^0^
16	1.85 × 10^11^± 4.95 × 10^10^	1.85 × 10^11^ ± 2.12 × 10^10^	2.65 × 10^11^ ± 4.95 × 10^10^	1.00 × 10^1^ ± 0.00 × 10^0^
20	4.80 × 10^12^ ± 2.83 × 10^11^	5.55 × 10^12^ ± 6.36 × 10^11^	1.21 × 10^1^^3^ ± 4.10 × 10^12^	1.00 × 10^1^ ± 0.00 × 10^0^

**Table A2:** Raw data of time kill analysis at 30 min interval for 8 h.

Time (h)	*K. pneumoniae* BAA-1705			
	Cell Number (CFU/mL) (Mean ± SD)			
	Control	CBO (0.08%)	Meropenem (16 µg/mL)	CBO (0.08%) + Meropenem (16 µg/mL)
0.0	2.25 × 10^4^ ± 3.54 × 10^3^	2.00 × 10^4^ ± 1.41 × 10^4^	2.70 × 10^4^ ± 4.24 × 10^3^	1.90 × 10^4^ ± 1.41 × 10^3^
0.5	2.50 × 10^4^ ± 7.07 × 10^3^	2.50 × 10^4^ ± 4.24 × 10^3^	2.45 × 10^4^ ± 3.54 × 10^3^	2.45 × 10^4^ ± 7.78 × 10^3^
1.0	2.90 × 10^4^ ± 4.24 × 10^3^	2.70 × 10^4^ ± 4.24 × 10^3^	6.50 × 10^3^ ± 7.07 × 10^2^	7.00 × 10^3^ ± 1.41 × 10^3^
1.5	4.90 × 10^4^ ± 1.41 × 10^3^	3.05 × 10^4^ ± 6.36 × 10^3^	3.50 × 10^3^ ± 7.07 × 10^2^	1.00 × 10^1^ ± 0.00 × 10^0^
2.0	7.75 × 10^4^ ± 7.07 × 10^2^	6.90 × 10^4^ ± 1.41 × 10^3^	1.50 × 10^3^ ± 7.07 × 10^2^	1.00 × 10^0^ ± 0.00 × 10^0^
2.5	7.60 × 10^5^ ± 5.66 × 10^4^	2.40 × 10^5^ ± 8.49 × 10^4^	1.00 × 10^1^ ± 0.00 × 10^0^	1.00 × 10^1^ ± 0.00 × 10^0^
3.0	5.20 × 10^6^ ± 2.83 × 10^5^	1.60 × 10^6^ ± 8.49 × 10^5^	1.00 × 10^1^ ± 0.00 × 10^0^	1.00 × 10^1^ ± 0.00 × 10^0^
3.5	1.80 × 10^7^ ± 2.83 × 10^6^	3.25 × 10^6^ ± 6.36 × 10^5^	1.00 × 10^1^ ± 0.00 × 10^0^	1.00 × 10^1^ ± 0.00 × 10^0^
4.0	9.05 × 10^7^ ± 1.06 × 10^7^	1.35 × 10^7^ ± 2.12 × 10^6^	1.00 × 10^1^ ± 0.00 × 10^0^	1.00 × 10^1^ ± 0.00 × 10^0^
4.5	1.65 × 10^8^ ± 4.95 × 10^7^	2.20 × 10^7^ ± 5.66 × 10^6^	1.00 × 10^1^ ± 0.00 × 10^0^	1.00 × 10^1^ ± 0.00 × 10^0^
5.0	3.65 × 10^8^ ± 6.36 × 10^7^	2.60 × 10^7^ ± 5.66 × 10^6^	1.75 × 10^6^ ± 3.54 × 10^5^	1.00 × 10^1^ ± 0.00 × 10^0^
5.5	5.90 × 10^8^ ± 1.41 × 10^7^	8.80 × 10^7^ ± 5.66 × 10^6^	9.50 × 10^6^ ± 7.07 × 10^5^	1.00 × 10^1^ ± 0.00 × 10^0^
6.0	6.80 × 10^8^ ± 2.83 × 10^7^	3.90 × 10^8^ ± 1.41 × 10^7^	4.20 × 10^8^ ± 1.70 × 10^8^	1.00 × 10^1^ ± 0.00 × 10^0^
6.5	6.30 × 10^9^ ± 9.90 × 10^8^	5.20 × 10^8^ ± 1.13 × 10^8^	1.05 × 10^9^ ± 7.07 × 10^7^	1.00 × 10^1^ ± 0.00 × 10^0^
7.0	7.55 × 10^9^ ± 6.36 × 10^8^	1.14 × 10^9^ ± 2.26 × 10^8^	1.75 × 10^9^ ± 6.36 × 10^8^	1.00 × 10^1^ ± 0.00 × 10^0^
7.5	2.50 × 10^10^ ± 7.07 × 10^9^	4.55 × 10^9^ ± 6.36 × 10^8^	1.60 × 10^10^ ± 5.66 × 10^9^	1.00 × 10^1^ ± 0.00 × 10^0^
8.0	8.45 × 10^10^ ± 7.78 × 10^9^	2.35 × 10^10^ ± 9.19 × 10^9^	6.55 × 10^10^ ± 6.36 × 10^9^	1.00 × 10^1^ ± 0.00 × 10^0^

**Table A3:** Raw data of zeta potential measurement.

***K. pneumoniae* BAA-1705**	**Zeta Potential (mV) (Mean ± SD)**			
	**Control**	**Cinnamon Bark**	**Meropenem**	**Cinnamon Bark + Meropenem**
	−11.10 ± 0.66	−3.27 ± 0.70	−3.72 ± 0.41	−2.62 ± 0.14

**Table A4:** Raw data of outer membrane permeability assay.

Time	Treatment OD_600nm_ ± SD (n = 3)							
	Control		CBO (0.08%)		Meropenem (16 µg/mL)		CBO (0.08%) + Meropenem (16 µg/mL)	
	Without 0.1% SDS	With 0.1% SDS	Without 0.1% SDS	With 0.1% SDS	Without 0.1% SDS	With 0.1% SDS	Without 0.1% SDS	With 0.1% SDS
**0**	0.68 ± 0.027	0.68 ± 0.006	0.50 ± 0.016	0.49 ± 0.010	0.72 ± 0.025	0.66 ± 0.016	0.50 ± 0.017	0.45 ± 0.017
**5**	0.72 ± 0.047	0.70 ± 0.002	0.51 ± 0.007	0.46 ± 0.003	0.75 ± 0.005	0.66 ± 0.011	0.53 ± 0.008	0.43 ± 0.004
**10**	0.90 ± 0.007	0.76 ± 0.008	0.62 ± 0.015	0.50 ± 0.006	0.89 ± 0.013	0.72 ± 0.006	0.52 ± 0.007	0.42 ± 0.021
**30**	1.32 ± 0.005	1.05 ± 0.007	1.04 ± 0.004	0.67 ± 0.008	1.25 ± 0.021	1.02 ± 0.021	0.51 ± 0.015	0.40 ± 0.014
**60**	1.66 ± 0.007	1.37 ± 0.009	1.37 ± 0.004	0.92 ± 0.004	1.56 ± 0.031	1.34 ± 0.011	0.53 ± 0.015	0.38 ± 0.009
